# Neuropeptide-Driven Cross-Modal Plasticity following Sensory Loss in *Caenorhabditis elegans*

**DOI:** 10.1371/journal.pbio.1002348

**Published:** 2016-01-08

**Authors:** Ithai Rabinowitch, Patrick Laurent, Buyun Zhao, Denise Walker, Isabel Beets, Liliane Schoofs, Jihong Bai, William R. Schafer, Millet Treinin

**Affiliations:** 1 Department of Medical Neurobiology, Hadassah Medical School, Hebrew University of Jerusalem, Jerusalem, Israel; 2 Neurobiology Division, MRC Laboratory of Molecular Biology, Cambridge, United Kingdom; 3 Basic Sciences Division, Fred Hutchinson Cancer Research Center, Seattle, Washington, United States of America; 4 ULB Neuroscience Institute, Université Libre de Bruxelles, Bruxelles, Belgium; 5 Functional Genomics and Proteomics, KU Leuven, Leuven, Belgium; Brandeis, UNITED STATES

## Abstract

Sensory loss induces cross-modal plasticity, often resulting in altered performance in remaining sensory modalities. Whereas much is known about the macroscopic mechanisms underlying cross-modal plasticity, only scant information exists about its cellular and molecular underpinnings. We found that *Caenorhabditis elegans* nematodes deprived of a sense of body touch exhibit various changes in behavior, associated with other unimpaired senses. We focused on one such behavioral alteration, enhanced odor sensation, and sought to reveal the neuronal and molecular mechanisms that translate mechanosensory loss into improved olfactory acuity. To this end, we analyzed in mechanosensory mutants food-dependent locomotion patterns that are associated with olfactory responses and found changes that are consistent with enhanced olfaction. The altered locomotion could be reversed in adults by optogenetic stimulation of the touch receptor (mechanosensory) neurons. Furthermore, we revealed that the enhanced odor response is related to a strengthening of inhibitory AWC→AIY synaptic transmission in the olfactory circuit. Consistently, inserting in this circuit an engineered electrical synapse that diminishes AWC inhibition of AIY counteracted the locomotion changes in touch-deficient mutants. We found that this cross-modal signaling between the mechanosensory and olfactory circuits is mediated by neuropeptides, one of which we identified as FLP-20. Our results indicate that under normal function, ongoing touch receptor neuron activation evokes FLP-20 release, suppressing synaptic communication and thus dampening odor sensation. In contrast, in the absence of mechanosensory input, FLP-20 signaling is reduced, synaptic suppression is released, and this enables enhanced olfactory acuity; these changes are long lasting and do not represent ongoing modulation, as revealed by optogenetic experiments. Our work adds to a growing literature on the roles of neuropeptides in cross-modal signaling, by showing how activity-dependent neuropeptide signaling leads to specific cross-modal plastic changes in neural circuit connectivity, enhancing sensory performance.

## Abstract 2

Touch-sensing neurons in the nematode *Caenorhabditis elegans* secrete neuropeptides to suppress olfaction via a specific synapse in the olfactory circuit. Loss of touch releases this suppression, enhancing the sense of smell.

## Author Summary

The brain has the remarkable capacity to respond to sensory loss by boosting remaining functioning senses. For example, certain features of hearing are improved in blind people. What are the cellular and molecular mechanisms underlying this effect? How is a certain sense strengthened? If it is possible to hear better, why don’t we hear better in the first place? To simplify these problems, we examined them in an organism with a substantially less complicated nervous system than our own, the roundworm *C*. *elegans*. We discovered that *C*. *elegans* mutants that cannot sense touch to the body exhibit an improved sense of smell. We were able to pinpoint this change in sensory performance to a change in strength of a specific synapse in the olfactory circuit. We further found that in normal worms, this olfactory synapse is suppressed through a neuropeptide signal transmitted from the touch sensing neurons. In contrast, without any touch input, the touch neurons secrete less neuropeptide, the olfactory synapse becomes stronger, and the sense of smell improves. We were able to reverse these effects by artificially stimulating the touch neurons and by engineering a new synapse into the olfactory circuit.

## Introduction

Sensory loss often elicits cross-modal plasticity, either enhancing or reducing the performance of remaining unimpaired sensory modalities. These effects have been broadly described in humans and other mammals [1,2] and exemplify the remarkable plasticity and adaptability of the brain. What drives cross-modal plasticity and how this influences sensory performance has been mainly addressed at the macroscopic level of entire brain structures [3,4]. For example, it has been shown that in the blind, the visual cortex is recruited to process various auditory features [5,6], and at the same time the auditory cortex may expand its tonotopic area [7] or exhibit changes in its functional responses [8]. Such system-wide neuroplasticity might stem directly from the silencing of neurons and neural circuits associated with the dysfunctional sensory modality, leading, for example, to reduced competition for neural targets [9]. Additionally, cross-modal plasticity might result also from an increased use-dependent plasticity [10] of the remaining functioning senses, or from an increase in the attention directed towards them [11]. These types of plasticity do not require activity-dependent signaling between modalities.

Whereas cross-modal plasticity has been largely studied at the system level of entire brain regions, much less is known about its cellular and molecular underpinnings. Recent work is just beginning to address this. For example, two recent studies have revealed prominent strengthening of α-amino-3-hydroxy-5-methyl-4-isoxazolepropionic acid (AMPA) receptor-mediated synaptic transmission in pyramidal neurons of layer 2/3 barrel cortex of visually-deprived rats [11,12]. These changes depended on long-distance serotonin signaling, presumably originating from the raphe nuclei [11]. Conversely, sensory deprivation of neonatal mice was shown to down-regulate oxytocin neuropeptide secretion from the hypothalamus, resulting in decreases in synaptic transmission in sensory cortical regions associated with nondeprived sensory modalities [13]. Both mechanisms rely on long-distance signaling. Interestingly, recently, long-distance neuropeptide and hormone signaling from one sensory modality was shown to modulate concurrent sensory responses in another modality in *C*. *elegans* [14–16]. We thus asked whether, in *C*. *elegans*, long-range signaling also leads to long-lasting plastic changes in sensory acuity.

For this purpose, we examined *C*. *elegans* mechanosensory (Mec) mutants lacking a sense of gentle touch to the body, and observed various changes in behaviors that depend on remaining senses. One notable alteration was enhanced chemosensation, an increased response to low concentrations of certain attractive odors. This suggests that cross-modal sensory compensation following sensory loss is a basic and conserved feature of the nervous system not limited to the complex brains of mammals. In particular, we were able to identify a specific synapse in the chemosensory circuit that is modulated by neuropeptide secretion from the Mec touch receptor neurons (TRNs) to tune chemosensory performance and to thus implement a form of cross-modal compensatory plasticity in *C*. *elegans*. To our knowledge, such neuropeptide-mediated synaptic plasticity has not been described before in *C*. *elegans*.

## Results

### Loss of Body Touch Leads to Enhanced Olfaction

**Fig 1:**
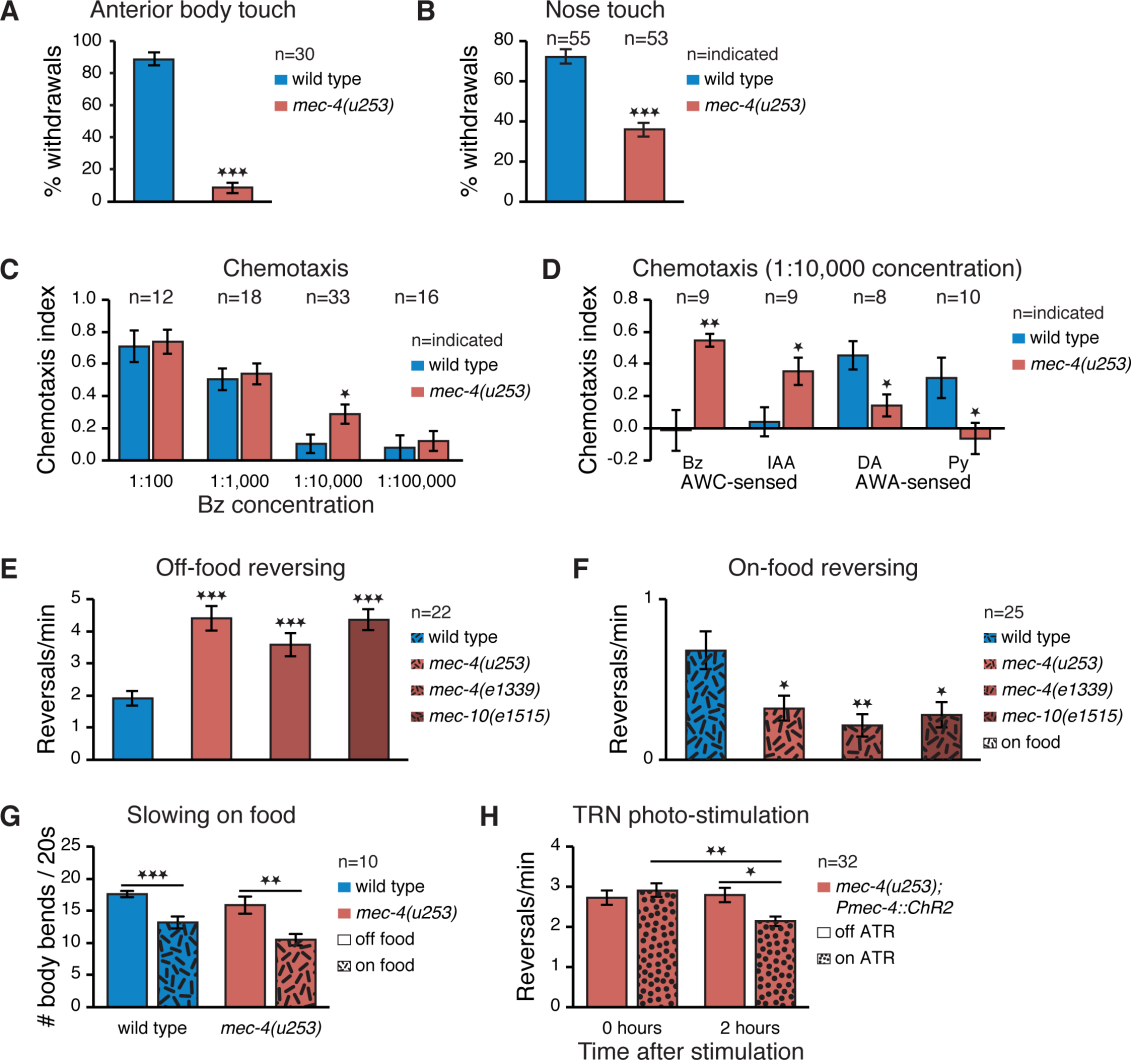
Loss of body touch response results in activity-dependent changes in locomotion and chemotaxis. (A) Percent of withdrawal responses out of five anterior body gentle stimulations of wild-type an Mec mutants. (B) Percent of withdrawal responses out of five gentle nose stimulations of wild type and Mec mutants. Worms defective in sensing body touch also show reduced nose touch responses. (C) Chemotaxis scores of wild type and Mec mutants at varying concentrations of the attractive odorant, Bz. A score of 1 corresponds to perfect attraction; a score of 0 indicates complete indifference. mec-4(u253) mutants outperform wild type at low, 1:10,000, Bz concentrations. (D) Chemotaxis scores of wild type and Mec mutants for 1:10,000 concentrations of AWC-sensed Bz and IAA, and AWA-sensed DA and Py. Chemosensation of AWC-sensed odors is enhanced. Chemosensation of AWA-sensed odors is reduced. (E,F) Reversing frequency off-food (E) and on-food (F) of various Mec mutants. Reversing off-food is increased and on-food is decreased in Mec mutants compared to wild type. (G) Speed off-food and on-food of wild type and Mec mutants. Wild type and Mec mutants exhibit similar slowing on food (2-way ANOVA interaction *p* = 0.6, main effect of food *p* < 0.0001). (H) Mec mutant reversing rate off-food immediately or 2 h after artificial stimulation of the TRNs using random blue light flashing, with or without (control) supplemental all-trans retinal (ATR). TRN stimulation reduced the reversing rate of mec-4(u253) mutants towards wild type levels 2 hours later (2-way ANOVA interaction *p* = 0.014). N2 is the wild type strain. Sample size indicated in each panel; Error bars represent standard errors of the mean (SEMs); *p < 0.05, **p < 0.01, ***p < 0.001 *t* test with Bonferroni corrections for multiple comparisons where relevant.

In order to identify cross-modal plasticity following sensory loss in *C*. *elegans*, we focused on the Mec-deficient mutant, *mec-4(u253)*, which lacks functional MEC-4, a DEG/ENaC channel subunit exclusively expressed in the TRNs and necessary for sensing gentle touch to the body (Fig 1A) [17–19]. We first examined the *mec-4* response to nose touch [20] and found that, even though body and nose touch are mediated by distinct sensory neurons and mechanoreceptors [21], loss of body touch leads to reduced nose touch (Fig 1B). We also tested *mec-4(u253)* chemosensation [22] by performing a chemotaxis assay using the attractive odorant benzaldehyde (Bz). We found that specifically at low Bz concentrations (1:10,000), the Mec mutants were more proficient in navigating towards the odor source than wild-type worms, as indicated by their higher chemotaxis index (Fig 1C). Bz is sensed by the AWC chemosensory neurons, which are also sensitive to isoamyl alcohol (IAA), another volatile attractant [23]. We found that similarly to Bz, chemotaxis to low concentrations of IAA was enhanced in *mec-4* worms (Fig 1D). In contrast, chemotaxis to diacetyl (DA) and pyrazine (Py), both of which are sensed by the AWA chemosensory neurons [23], was attenuated in *mec-4* (Fig 1D). These data illustrate two forms of cross-modal plasticity following sensory loss in *C*. *elegans*, one enhancing (Bz and IAA chemotaxis) and one reducing (nose touch, DA and Py chemotaxis) remaining sensory responses. In the present study, we focus on cross-modal sensory enhancement, as exhibited by an augmented AWC-mediated olfactory acuity in touch-deficient worms.

### Enhanced Olfaction Correlates with Food-Dependent Changes in Locomotion in Touch Insensitive Worms

*C*. *elegans* chemotaxis is based on a biased random walk mechanism, whereby forward motion is interspersed with randomly occurring reorienting reversals and turns, whose frequency varies as a function of odor concentration [24,25]. This produces a net movement up chemical gradients towards the source of an attractant. As a consequence, animals removed from their food transiently increase their reversing rate compared to animals on food, since they sense a decrease in odor concentration. Thus, an alternative assay for olfaction consists of measuring the increased reversing rate of animals just removed from their source of food. This response, which like chemotaxis itself has been shown to be controlled by the AWC olfactory circuit [26–28], thus can serve as a more sensitive measure for analyzing chemotaxis in individuals or small groups of animals [28].

We thus compared the frequency of reversing off and on food between wild-type and Mec mutants. For these experiments we tested two independent *mec-4* alleles with full or partial loss of body touch sensitivity, *mec-4(u253)* and *mec-4(e1339)*, respectively, as well as a *mec-10(e1515)* mutant, with dysfunctional MEC-10, a subunit like MEC-4 of the DEG/ENaC mechanosensory channel complex [29]. The off-food reversing rate of all Mec mutants was significantly higher than that of wild type (Fig 1E) and significantly lower than wild type on-food reversing rate (Fig 1F), suggesting that locomotion of Mec mutants is indeed more sharply tuned to the presence or absence of food cues, as sensed by the AWC olfactory circuit [28,30].

In addition to chemosensory and olfactory neurons, food is sensed also by a group of dopaminergic mechanosensory neurons, which are distinct from the TRNs, and mediate a slowing in speed in the presence of food [31]. We tested whether the food-dependent changes in reversing rate in Mec mutants (Fig 1E and 1F) might be associated with improper mechanical food sensing, but found normal slowing on food in *mec-4* mutants (Fig 1G), ruling out this possibility.

### Altered Reversing Rate Is Activity-Dependent and Development-Independent

We asked whether the altered reversing rate of *mec-4* mutants is associated with reduced TRN activity. We used an optogenetic approach to address this question. We artificially activated the TRNs of *mec-4* and tested whether this manipulation would reduce reversing rate back towards normal. We did this by expressing Channelrhodopsin2 (ChR2) specifically in the TRNs of *mec-4* worms using the *mec-4* promoter and stimulated the TRNs with random flashes of blue light for a period of 80 min. Since *C*. *elegans* must be fed all-trans retinal (ATR) in order for ChR2 to be activated by light [32], we compared the post flashing reversing rate of adult worms that had or had not been fed with ATR and found a significant decrease in reversing rate 2 h, but not immediately, after photo-stimulation in worms exposed to ATR (Fig 1H). This result indicates that the cross-modal change observed in Mec mutants is enduring and depends on the history of TRN activity rather than on ongoing or recent activity. It also demonstrates that this form of cross-modal plasticity does not depend on developmental effects, since it was readily reversible in adults.

### Enhanced AWC→AIY Inhibitory Synaptic Transmission in Touch Insensitive Worms

**Fig 2:**
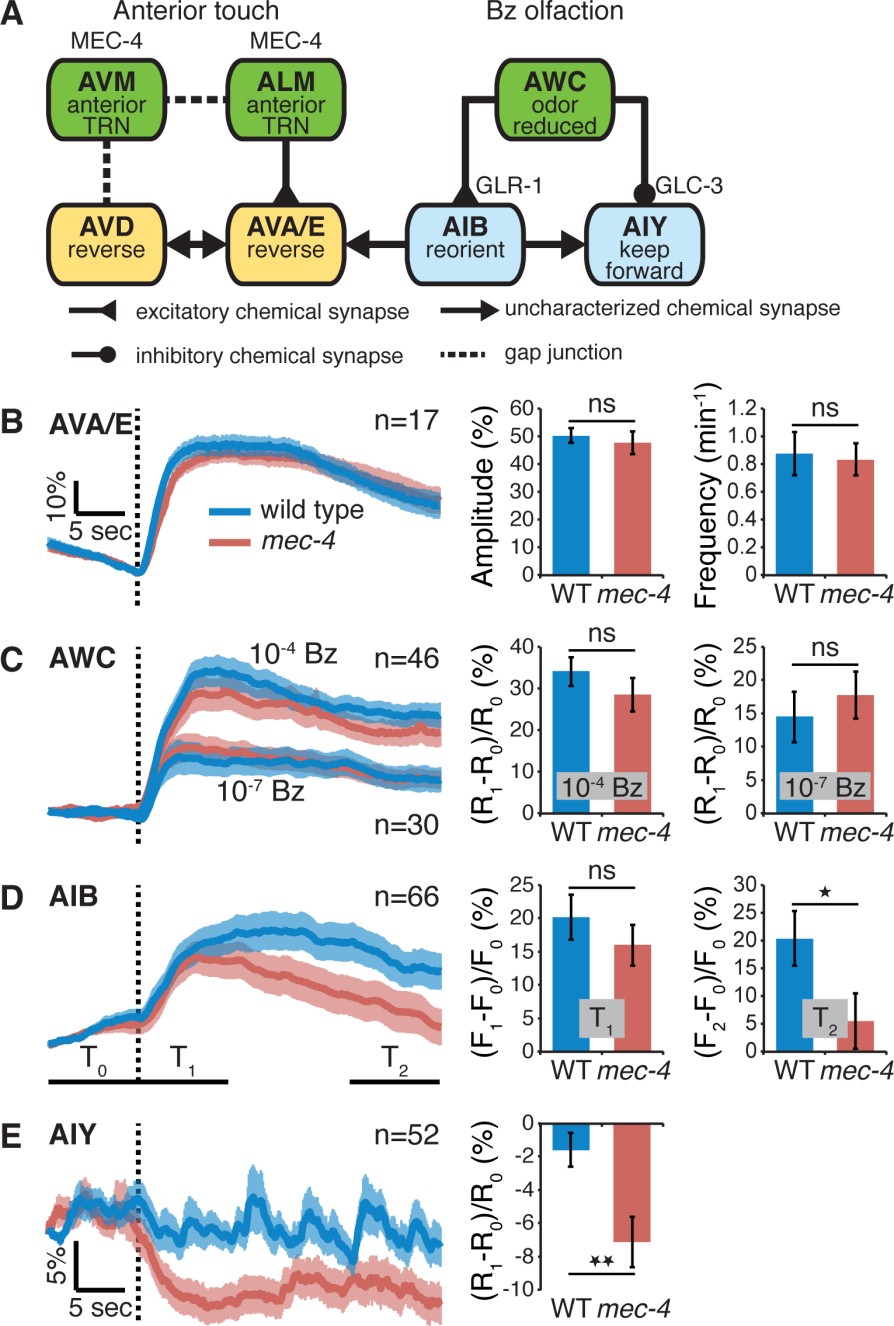
Loss of body touch results in increased inhibitory transmission between AWC and AIY. (A) Principal neurons comprising the body touch mechanosensory circuit (left) and Bz-sensing olfactory circuit (right). Indicated are synapse types and synaptic receptors in AIB and AIY. (B) Averaged trace (left), amplitude (middle), and frequency (right) of spontaneous calcium transients recorded in AVA/E. (C–E) Averaged trace (left) and mean ratio (C and E) or fluorescence (D) change (middle and right) before and after 1:10,000 Bz removal (dotted line) recorded in AWC (C), AIB (D), and AIY (E) neurons in wild-type (N2) and *mec-4(u253)* mutants. AWC (C) was also tested with a lower concentration of Bz (1:10,000,000; ratio change—right). T0, T1, and T2 in D indicate the averaging windows for computing fluorescence: F0 and R0 are fluorescence, and ratio change averaged over T0. F1 and R1 are averages over T1. F2 is the average over T2. The AIB response to Bz removal was attenuated (D). The AIY response to Bz removal was augmented (E). Sample size is indicated in each panel; Error bars represent SEMs; *p < 0.05, **p < 0.01 *t* test.

We next sought to identify the neural mechanisms underlying cross-modal compensatory behavior between touch and smell (olfaction), by comparing the activity patterns of neurons involved in the mechanosensory or AWC-associated olfactory networks in wild type relative to *mec-4(u253)* mutants. To this end, we performed calcium-imaging experiments using microfluidic devices [33]. We first considered a subset of premotor interneurons, AVA and AVE, which control, in part, reversing behavior [34–36], and that have direct synaptic connections with the touch receptor neurons (Fig 2A) [17,37]. Previous work has shown that spontaneous calcium transients in these neurons correspond to spontaneous reversing behavior [33,38]. We thus expected *mec-4(u253)* AVA/AVE neurons to exhibit enhanced activity in the absence of food compared to wild type, if they are involved in increasing reversing behavior. We found, however, no differences between AVA/E wild type and *mec-4(u253)* averaged spontaneous calcium transient traces, their amplitude, or frequency (Fig 2B). Since the additional reversal-promoting premotor neuron pair, AVD, seems to be mainly involved in orchestrating touch-evoked withdrawals rather than spontaneous reversing [17,39,34], we did not attempt to image spontaneous activity in this neuron.

Next, we considered neurons involved in Bz chemotaxis (Fig 2A). Bz, IAA, and other odorants are detected by the AWC pair of sensory neurons, which increase their activity as odor concentrations decrease [28]. We expected that if AWC neurons contribute to enhancing off-food reversing rate and chemotaxis following loss of mechanosensation by increasing their sensitivity, then they should show a larger response to reduced odor concentration in the Mec mutants. However, we found no significant difference in the AWC response to Bz (1:10,000) removal between wild type and *mec-4(u253)* animals (Fig 2C). This was also true for a considerably lower concentration of Bz (1:10,000,000; Fig 2C, right), indicating that the similar AWC responses of wild type and *mec-4(u253)* mutants are not likely due to some limit in the AWC or calcium sensor dynamic range.

The AWC sensory neurons make excitatory synaptic connections with a pair of interneurons, AIB, which promote reversing when active (Fig 2A) [25,28]. Thus, for AIB to be considered as a source for enhanced reversing and chemotaxis, it should display an enlarged response to odor removal in Mec-deficient worms compared to wild type. In effect, the initial calcium responses of AIB to Bz removal were similar in wild type and *mec-4(u253)* (Fig 2D). Moreover, the AIB response in *mec-4(u253)* animals decayed more rapidly (beginning approximately 5 sec after onset; Fig 2D), entailing overall reduced rather than enhanced AIB activity, which, if at all, should reduce and not enhance reversing. This delayed effect might be due to some negative feedback mechanism within the olfactory circuit, perhaps similar to other neuropeptide-dependent feedback loops already shown to act in this circuit [40].

The AWC odor-sensing neurons also make inhibitory synapses with the AIY interneuron pair (Fig 2A) [28]. Artificial inhibition of AIY activity has been directly shown to enhance reversing [41]. Thus, if the AIY neurons are involved in enhancing off-food reversing in Mec mutants, then they should respond with a larger inhibition following odor removal. Indeed, *mec-4(u253)* worms exhibited more prominent AIY negative calcium responses than wild type (Fig 2E). The enlarged AIY inhibitory response in the Mec mutants (Fig 2E) on the one hand, and the similarity in the AWC and initial AIB responses between wild type and *mec-4(u253)* worms (Fig 2C and 2D) on the other hand, suggest together that the enhanced acuity of Mec-deficient worms to Bz might stem from potentiated AWC→AIY inhibitory transmission.

#### Altered AWC→AIY synaptic transmission affects reversing rate in Mec mutants

**Fig 3:**
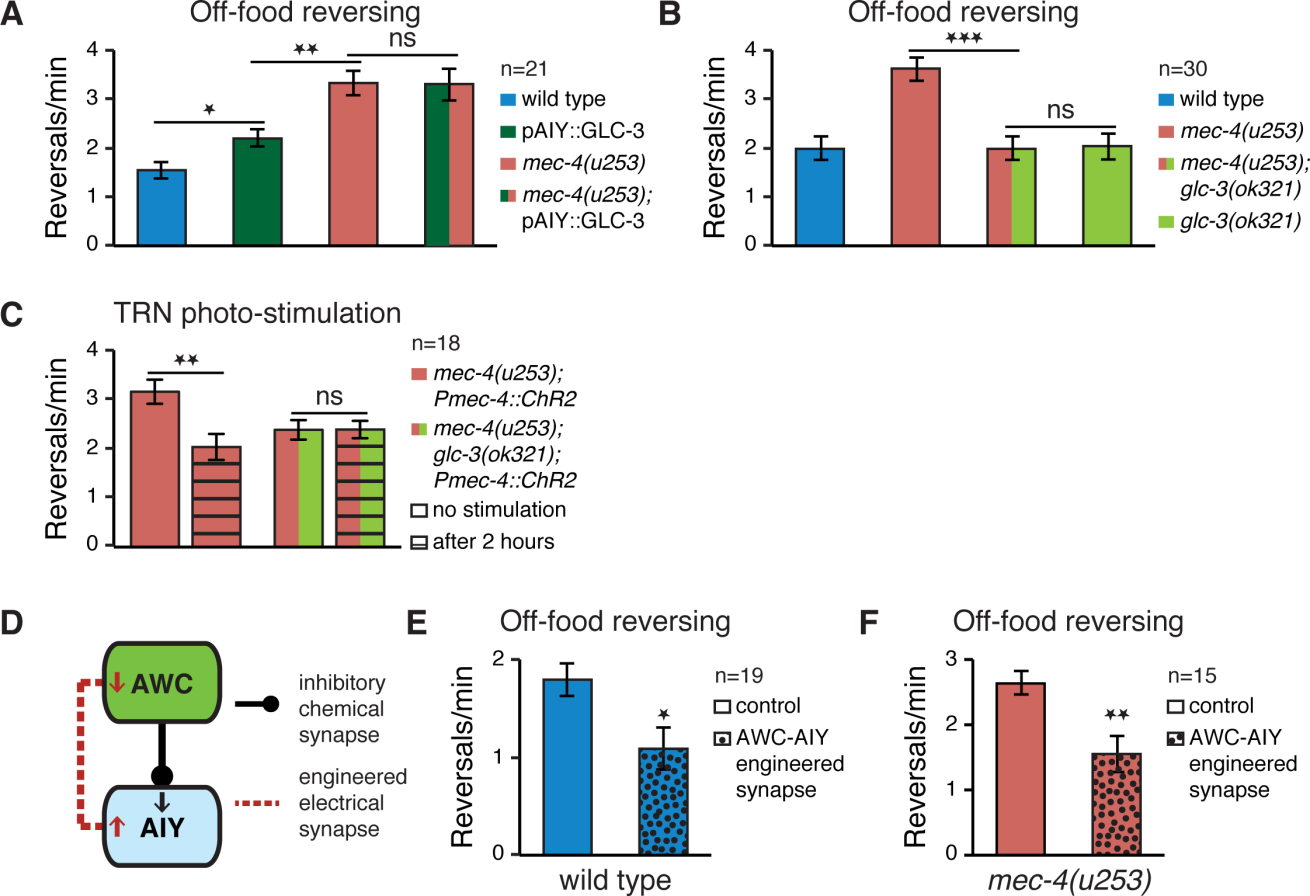
AWC→AIY transmission underlies TRN activity-dependent modulation of locomotion. (A) Overexpression of glutamate receptor GLC-3 in AIY increases off-food reversing rate, but is not additive with the effects of loss of body touch. (B) In *glc-3* mutants, loss of touch responsiveness does not increase reversing rate. (C) Optogenetic TRN stimulation of *glc-3; mec-4* double mutants does not alter their reversing frequency off-food compared to naïve controls (2-way ANOVA interaction *p* = 0.014). (D) Inserting an engineered electrical synapse between AWC and AIY attenuates AWC→AIY inhibitory transmission, since it offsets the AWC to AIY inhibitory negative signal (↓) with a positive signal (↑) and at the same time also feeds back a negative signal (↓) into AWC. (E) An engineered electrical synapse inserted between AWC and AIY reduces reversing frequency in wild type. (F) An engineered electrical synapse inserted between AWC and AIY counteracts the increased reversing rate of Mec mutants. Sample size indicated in each panel; Error bars represent SEMs; *p < 0.05, **p < 0.01, ***p < 0.001 *t* test with Bonferroni corrections for multiple comparisons where relevant.

AWC→AIY synaptic transmission consists of glutamate release from AWC, and the subsequent opening of GLC-3 containing glutamate-gated chloride channels in AIY [28]. Overexpression of GLC-3 in AIY has been previously shown to enhance off-food reversing rate [28]. We wished to examine the effects of such GLC-3 overexpression on *mec-4(u253)* mutants. We found no further increase in reversing rate between *mec-4(u253)* mutants alone and *mec-4(u253)* mutants overexpressing GLC-3 in AIY (Fig 3A). The lack of additivity between *mec-4(u253)* and GLC-3 overexpression suggests that their effect on off-food reversing frequency originates from the same pathway. This lack of additivity is not likely due to a ceiling on reversing frequency since other strains exist that exhibit a substantially higher reversing rate than what we observed for *mec-4* mutants [35]. A double mutant combining *mec-4(u253)* and *glc-3(ok321)*, a *glc-3* deletion mutant, suppressed the increased *mec-4(u253)* reversing rate (Fig 3B). Moreover, the reversing rate of *glc-3(ok321)* worms was similar in *mec-4(u253)* and Mec-normal worms (Fig 3B), indicating that GLC-3 is likely to function downstream of *mec-4(u253)* in its effect on off-food reversing frequency.

We examined whether restoring the reversing rate of *mec-4(u253)* by TRN photo-stimulation depends on functional GLC-3 receptors. Unlike *mec-4(u253)*, photo-stimulated *mec-4(u253); glc-3(ok321)* double mutants expressing ChR2 in their TRNs showed no change in reversing frequency compared to nonstimulated controls (Fig 3C), indicating that TRN activity-dependent changes in reversing rate require functional GLC-3.

Although GLC-3 overexpression or removal affects AWC→AIY transmission, this receptor might also be involved in synaptic signaling to AIY originating from other neurons. We have recently shown that inserting an electrical synapse between AWC and AIY, by expressing in both neurons the mouse gap junction protein connexin 36 (Cx36) diminishes and may even invert AWC→AIY inhibition [42]. The reason is that electrical synapses, unlike chemical synapses, cannot reverse an excitatory signal and thus negate or reduce the inhibitory effects of the chemical AWC→AIY synapse. At the same time, they also reduce AWC excitability by shunting current from AWC to AIY, decreasing the degree of inhibitory transmission (Fig 3D). In particular, we have demonstrated that inserting an engineered AWC–AIY electrical synapse in the olfactory circuit completely abolishes chemotaxis to Bz [42]. In order to test whether specific suppression of the AWC→AIY synapse is sufficient also for reducing reversing rate, we compared between worms with and without an engineered AWC–AIY electrical synapse. Indeed, inserting an electrical synapse between AWC and AIY caused a decrease in reversing frequency in wild type (Fig 3E). It was also sufficient for counteracting the enhanced reversing of *mec-4(u253)* mutants (Fig 3F).

Taken together, the imaging results, the genetic analysis, and the synaptic engineering experiments in Figs 2 and 3 suggest that defective mechanosensation leads to an increase in the inhibitory AWC→AIY signaling, which alters the frequency of off-food reversing and modifies chemotaxis performance.

### Reduced TRN-Secreted Neuropeptide Signaling Alters Reversing Rate

**Fig 4:**
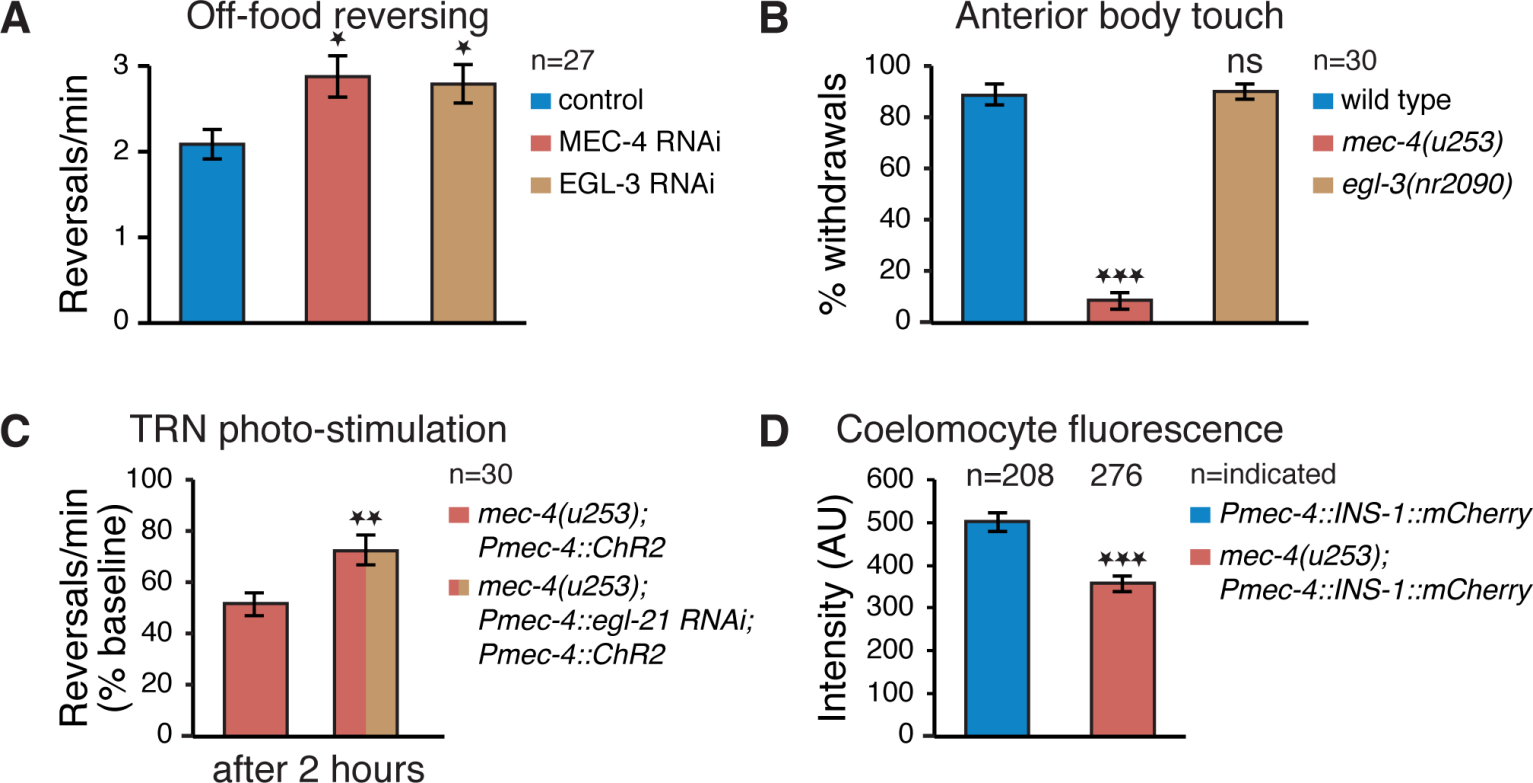
TRN neuropeptide signaling alters the reversing rate of Mec mutants. (A) Off-food reversing rate increases following TRN-specific RNAi silencing of MEC-4 and EGL-3. (B) Percent of withdrawal responses out of five anterior body gentle stimulations of wild type, *mec-4* and *egl-3* mutants. *egl-3* mutants respond similarly to wild type. (C) Suppression of *mec-4* off-food elevated reversing rate following TRN optogenetic stimulation is diminished by TRN-specific silencing of EGL-21. (D) Coelomocyte fluorescence of mCherry-tagged insulin-like peptide transgene (INS-1) specifically expressed in the TRNs is decreased in *mec-4* mutants. Sample size indicated in each panel; Error bars represent SEMs; *p < 0.05, ***p < 0.001 *t* test with Bonferroni corrections for multiple comparisons where relevant.

Since there do not seem to be any direct synaptic connections between the TRNs and AWC or AIY (Fig 2A), we hypothesized that the strengthening of AWC→AIY and the ensuing enhanced reversing rate in Mec-deficient worms might depend on neuropeptide signaling, which does not require synaptic contact between neurons. Indeed, TRN-specific RNAi knockdown [43] of EGL-3, a proprotein convertase necessary for neuropeptide processing [44], resulted in an increased off-food reversing frequency similar to TRN-specific RNAi knockdown of MEC-4 (Fig 4A), supporting our hypothesis. To rule out the possibility that neuropeptide secretion might be necessary for mechanosensation itself, we compared the response to gentle body touch between wild type, *mec-4(u253)*, and *egl-3(nr2090)*. Only *mec-4(u253)* mutants displayed defective mechanosensation (Fig 4B). Notably, the *egl-3(nr2090)* responses were relatively small in magnitude, which might reconcile our results with previous accounts of Mec deficiency in *egl-3* mutants [44].

We also tested the effects of TRN-specific RNAi silencing [45] of EGL-21, a carboxypeptidase required for neuropeptide processing [46,47], on *mec-4(u253)* reversing rate 2 h post artificial (optogenetic) TRN activation. TRN photo-stimulation had a weaker effect on reversing rate (relative to baseline: the same strain without stimulation) upon TRN EGL-21 silencing compared to *mec-4(u253)* alone (Fig 4C), indicating that neuropeptide signaling from the TRNs contributes significantly to TRN activity-dependent changes in reversing rate. We note that the residual reduction in reversing may be a result of incomplete knockdown of EGL-21 by the *Pmec-4*::*egl-21* transgene.

In order to establish the impact of mechanosensory loss on TRN neuropeptide secretion, we performed a neuropeptide secretion imaging assay [48]. We measured coelomocyte fluorescence in worms expressing an mCherry-tagged insulin-like peptide transgene (INS-1) specifically in their TRNs. Since INS-1::mCherry is loaded together with all other neuropeptides in the cell into the same dense core vesicles, this assay does not segregate between different types of neuropeptides. *mec-4(u253)* mutants showed a reduced coelomocyte uptake of mCherry, implying a general decrease in neuropeptide secretion in *mec-4(u253)* mutants (Fig 4D).

### The FMRFamide-Related Neuropeptide FLP-20 Is Involved in Signaling from the TRNs Affecting Locomotion

**Fig 5:**
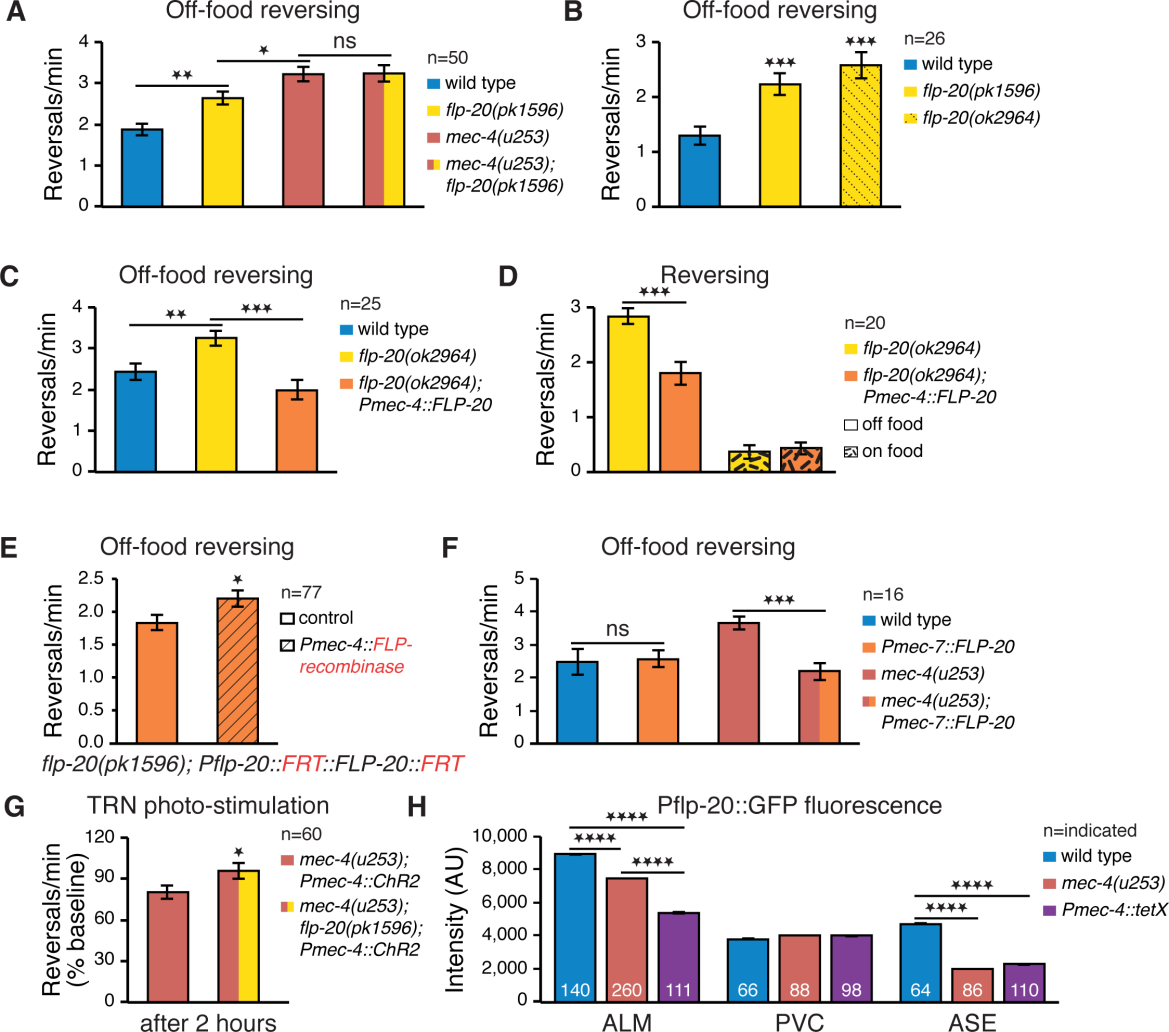
FLP-20 is involved in TRN neuropeptide signaling modulating locomotion. (A) *flp-20* mutants have an increased off-food reversing rate that is smaller than the *mec-4* increase. *flp-20; mec-4* double mutants exhibit a similar increase in off-food reversing rate as *mec-4* single mutants. (B) An additional *flp-20* allele displays a similar increase in reversing frequency. (C) TRN-specific expression of FLP-20 cDNA restores *flp-20* reversing rate off-food. (D) TRN-specific expression of FLP-20 cDNA reduces *flp-20* reversing rate off-food, but not on-food (2-way ANOVA interaction *p* = 0.0005). (E) Eliminating functional FLP-20 sequence exclusively in the TRNs is sufficient for increasing reversing rate. (F) Overexpression of FLP-20 in the TRNs of *mec-4(253)* mutants decreases their reversing rate. (G) TRN photo-stimulation has a weakened suppressive effect on the reversing rate of Mec-deficient worms lacking functional FLP-20. (H) Pflp-20::GFP fluorescence intensity, indicating FLP-20 transcription, in *mec-4(u253)* mutants and following disruption of exocytosis in the TRNs is reduced in ALM, does not substantially vary in PVC, and is also reduced in the ASE neurons (2-way ANOVA interaction *p* < 0.0001). Sample size indicated in each panel; Error bars represent SEMs; *p < 0.05, **p < 0.01, ***p < 0.001, ****p < 0.0001 *t* test with Bonferroni corrections for multiple comparisons where relevant.

Our results so far suggest that one or several neuropeptides expressed in the TRNs and processed by EGL-3 and EGL-21 convey the cross-modal plasticity observed following sensory loss. Recently, FLP-20, an FMRFamide-related neuropeptide expressed in the TRNs [49], has been shown to play a TRN-dependent role in mating behavior [50] and in short-term memory for mechanosensory habituation [51]. We wondered whether FLP-20 might also convey cross-modal plasticity following mechanosensory loss. To this end, we examined the reversing frequency of *flp-20(pk1596)* mutants, which harbor a deletion in their *flp-20* coding sequence. We found that, similarly to Mec mutants, *flp-20* worms show an increased reversing rate off-food (Fig 5A). Moreover, no differences were found in reversing frequency between *mec-4(u253)* mutants alone and *mec-4(u253); flp-20(pk1596)* double mutants (Fig 5A), suggesting that FLP-20 acts in the same pathway that produces enhanced reversing in *mec-4(u253)*. An additional allele, *flp-20(ok2964)*, displayed a similar increase in reversing compared to wild type (Fig 5B). Notably, although the reversing rate of *flp-20* mutants was higher than wild type, it was still lower than that of *mec-4* mutants, suggesting perhaps that additional neuropeptides might be involved in modulating reversing frequency off-food (Fig 5A). Transgenic expression of the FLP-20 transcript specifically in the TRNs reduced the enhanced *flp-20(ok2964)* reversing rate off-food (Fig 5C). To test whether this change in reversing rate is food-dependent, we compared the reversing rate of *flp-20* mutants and the TRN-specific rescue strain off-food and on-food (Fig 5D). We found a significant interaction between genotype and food (2-way ANOVA, F(1,76) = 13.30, *p* = 0.0005), indicating that TRN secretion of FLP-20 is important for modulating reversing rate in a food-dependent manner. Together, these results are consistent with a model whereby FLP-20 released from the TRNs diminishes the tuning of reversing rate to the abundance of food odor concentration.

To test whether elimination of FLP-20 specifically in the TRNs might increase reversing rate off-food, we constructed, using the Mos1 single-copy insertion (MosSCI) technique [52], a *flp-20(pk1596)* rescue strain carrying single-copy integrated FLP-20 driven by the FLP-20 promoter. We eliminated expression exclusively in the TRNs through cell-specific excision of the FLP-20 rescue sequence, using FLP-recombinase (see Materials and Methods). Reversing in this strain indeed increased following TRN-specific *flp-20* excision (Fig 5E), indicating that reduced FLP-20 signaling from the TRNs is sufficient to increase reversing rate. Conversely, overexpression of FLP-20 in the TRNs significantly suppressed the *mec-4(u253)* increase in reversing rate (Fig 5F); further indicating that FLP-20 is functionally released from the TRNs. It is noteworthy that the effectiveness of FLP-20 overexpression in *mec-4(u253)* mutants might suggest that the TRNs are still active, thus enabling neuropeptide release, even without mechanosensory input (e.g., due to spontaneous activity or input from other neurons). Further support for this is reported below (Fig 5H). We also tested whether the recovery of reversing rate in *mec-4(u253)* mutants following artificial photo-activation of the TRNs depended on FLP-20 signaling. We indeed found that the reduction in reversing post TRN stimulation was smaller in *mec-4(u253); flp-20(pk1596)* double mutants than in *mec-4(u253)* single mutants (Fig 5G), providing further evidence for the role of FLP-20 in modulating reversing rate as a function of TRN activity.

Above, we showed that reduced TRN activity due to loss of mechanosensation is associated with an overall decrease in neuropeptide secretion from the TRNs (Fig 4D). We wished to examine whether specifically FLP-20 signaling from the TRNs is reduced in Mec mutants. Recently, Laurent et al. introduced a new method for long-term monitoring of neurosecretion. The assay is based on the observation that reduced neurosecretion leads to reduced neuropeptide transcription, presumably via some feedback mechanism [53]. We thus measured ALM (one of the TRNs) and PVC (an interneuron also expressing FLP-20 but not MEC-4) fluorescence intensity in worms with an integrated *Pflp-20*::*GFP* array [54]. ALM but not PVC fluorescence in *mec-4(u253)* mutants was significantly decreased compared to wild type (Fig 5H), suggesting that attenuated TRN activity due to mechanosensory loss specifically reduces FLP-20 secretion from these neurons. We examined whether reduced TRN FLP-20 transcription was directly linked to diminished exocytosis. We did this by expressing tetanus toxin [55] in the TRNs, which cleaves synaptobrevin disrupting both clear and dense core vesicle release. This led to an even more pronounced decrease in Pflp-20::GFP fluorescence in ALM but not in PVC (Fig 5H), implying that indeed reduced neurosecretion in ALM correlates with reduced FLP-20 transcription in ALM, and that in spite of the loss of mechanosensation there might still be residual secretion of FLP-20 from the TRNs, consistent with the effectiveness of FLP-20 TRN overexpression (Fig 5F).

To ensure that mechanosensory loss does not confer a general change in transcription, we measured the ALM YFP fluorescence intensity of Pmec-4 driven YC2.12 (a calcium indicator composed of both YFP and CFP), in wild type and *mec-4(u253)* mutants treated with 0.01 M sodium azide (to eliminate YC2.12-sensed calcium fluctuations in these neurons). We found no significant difference in YFP expression between them (S1A Fig). To further examine the specificity of the *mec-*4 effect on FLP-20 release, we measured *Pflp-20*::*GFP* fluorescence in ASE neurons, which also express FLP-20. Whereas *mec-4(u253)*- and TRN-expressed tetanus toxin had no effect on PVC fluorescence, surprisingly, both caused a decrease in Pflp-20::GFP fluorescence in the ASE neurons (Fig 5H). This finding, which we leave for future investigation, suggests that the TRNs may modulate also ASE activity, and that in turn; in wild type worms the ASEs might relay and amplify FLP-20 signaling.

### FLP-20 Signaling Modulates the Olfactory Circuit

**Fig 6:**
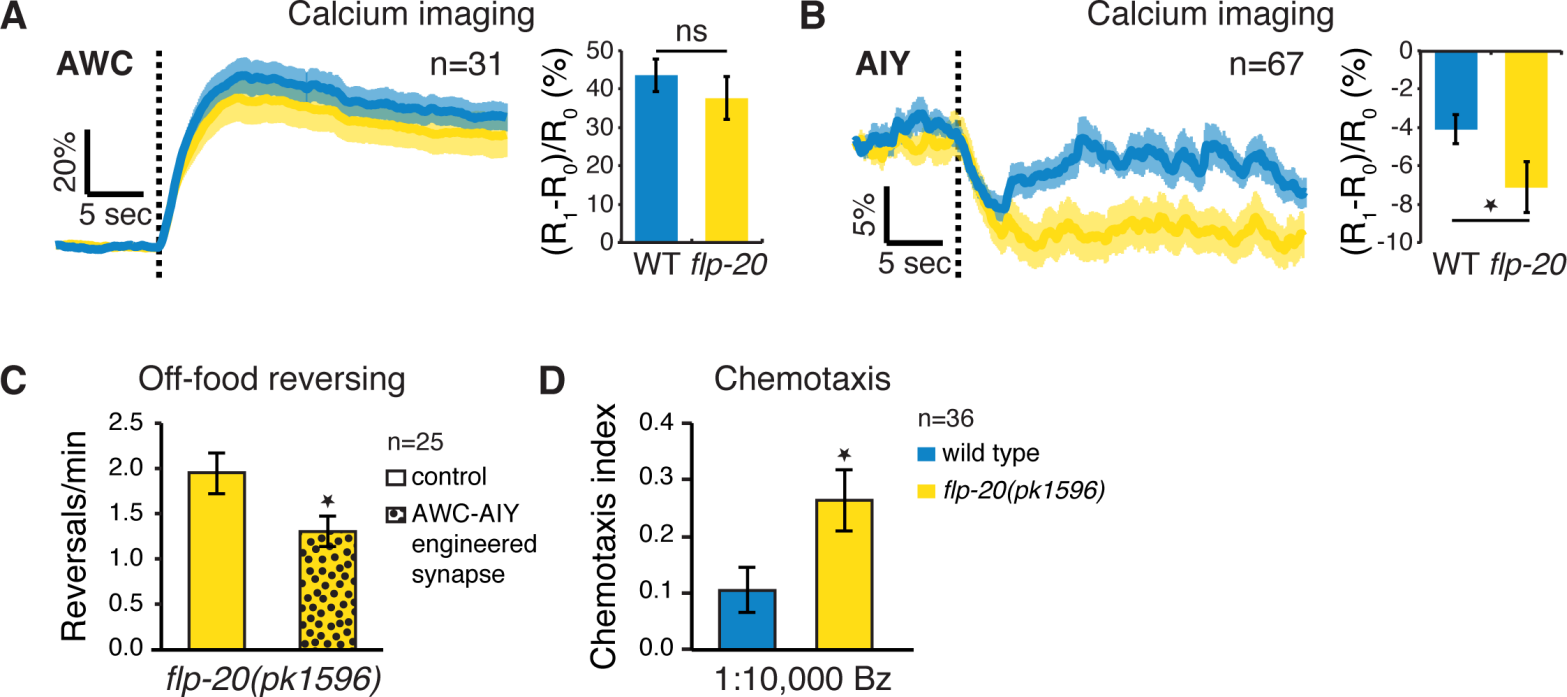
FLP-20 modifies AWC→AIY transmission regulating Bz chemotaxis. (A,B) Averaged trace (left) and mean ratio change (right) before and after Bz removal (dotted line) recorded in AWC (A) and AIY (B) neurons in wild type and *flp-20* mutants. The unchanged AWC response and the enhanced AIY response are similar to those of *mec-4* (Fig 2C and 2E). (C) An engineered electrical synapse inserted between AWC and AIY counteracts the increased reversing rate of *flp-20* mutants. (D) Chemotaxis to 1:10,000 Bz is enhanced in the absence of functional FLP-20. Sample size indicated in each panel; Error bars represent SEMs; *p < 0.05 *t* test.

As we have shown, FLP-20 signaling is correlated with TRN activity and can modify reversing rate. To determine whether it also affects AWC→AIY transmission, we measured calcium responses to Bz removal in wild type and *flp-20(pk1596)* mutants. As in *mec-4(u253)* mutants (Fig 2C and 2E), *flp-20(pk1596)* responses were similar to wild type in AWC (Fig 6A), but significantly enhanced in AIY (Fig 6B). Conversely, artificially reducing AWC→AIY inhibitory transmission by inserting an electrical synapse between AWC and AIY was sufficient to reduce *flp-20(pk1596)* reversing rate (Fig 6C), further confirming the link between FLP-20, reversing rate, and AWC→AIY synaptic transmission. Finally, we compared chemotaxis to low concentrations of Bz between wild type and *flp-20(pk1596)*, and found a significant increase in the chemotaxis index of worms lacking functional FLP-20 (Fig 6D), similar to the enhanced chemotaxis of Mec-deficient worms (Fig 1C and 1D). Taken together, these results are consistent with a simple model, whereby in Mec-deficient worms reduced neuropeptide secretion from the TRNs, including FLP-20, results in increased AWC→AIY inhibitory neurotransmission, leading to enhanced reversing off-food and AWC-dependent chemotaxis.

**Fig 7:**
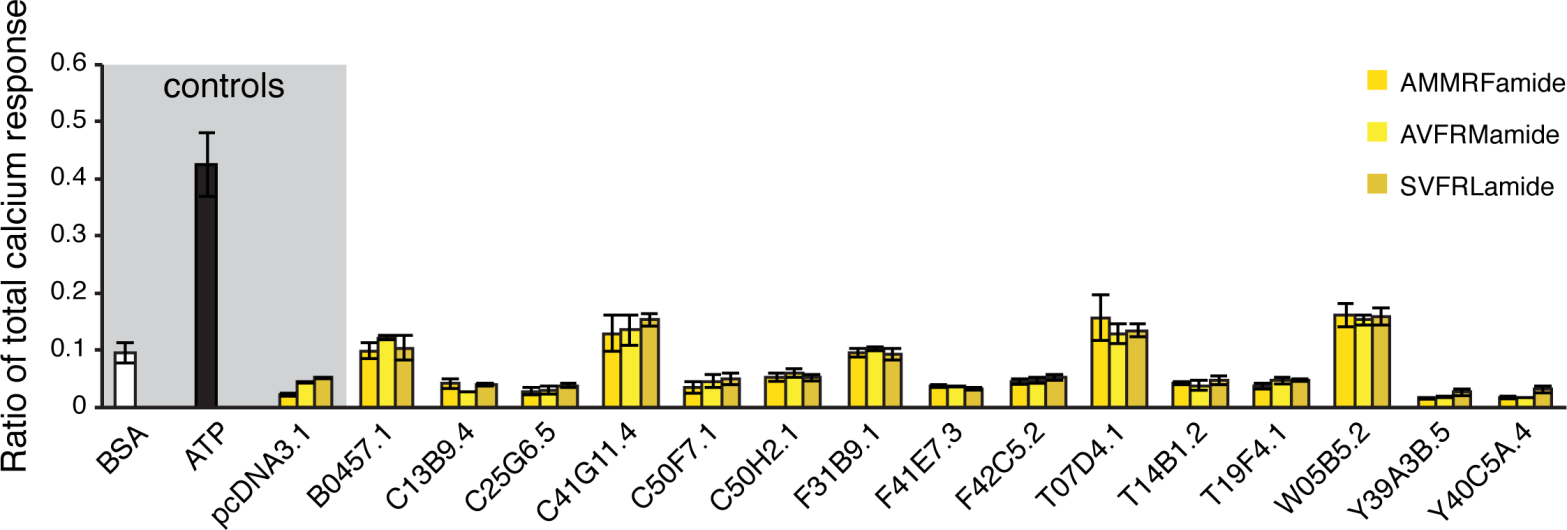
Screen for a FLP-20 AIY expressed receptor. Calcium responses measured in Chinese hamster ovary (CHO) cells transfected with an empty pcDNA3.1 vector or with a pcDNA3.1::receptor construct. Calcium responses after the administration of FLP-20 peptides are plotted as a ratio to the total calcium response (peptide-evoked + Triton-X-100- evoked responses). Bovine serum albumin (BSA) medium, containing no peptides, was used as a negative control. ATP, which activates an endogenous CHO receptor, was used as a positive control. BSA and ATP responses are averaged for all transfected constructs. Error bars indicate SEM (*n* ≥ 2).

Finally, we sought to identify the FLP-20 neuropeptide receptor, presumably acting in AIY to modulate AWC→AIY transmission. There is currently no known receptor for FLP-20, but several putative G-Protein Coupled Receptors are prominent in the AIY transcriptome [56]. We screened 15 candidate receptors using an aequorin assay [57] (see Materials and Methods) but none of them responded to neither of three FLP-20 derived peptides: AMMRFamide, AVFRMamide and SVFRLamide (Fig 7). Thus, some other FLP-20 receptor, yet to be determined, might act directly in AIY, or indirectly through another neuron.

## Discussion

**Fig 8:**
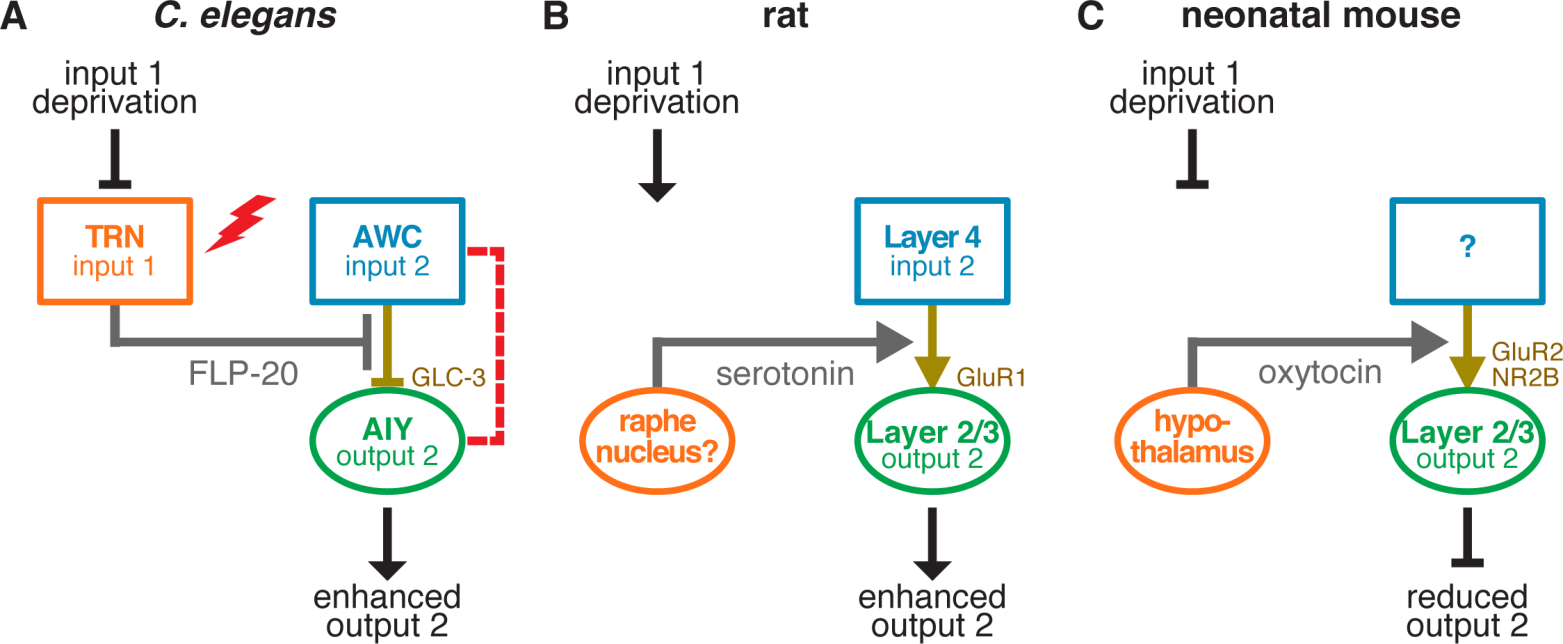
Molecular/cellular mechanisms for cross-modal plasticity following sensory loss. (A) Loss of body touch in *C*. *elegans* results in reduced neuropeptide secretion from the TRNs, leading to the release from suppression, i.e., strengthening, of the inhibitory glutamatergic synaptic connection between AWC chemosensory neurons and AIY interneurons and thus enhancing the output of the olfactory circuit. Optogenetic activation of the TRNs (red lightning bolt) or insertion of an engineered electrical synapse between AWC and AIY (red dashed line) can counteract these effects. (B) Visual deprivation in rats results in increased serotonin signaling, possibly from the raphe nucleus, increasing extracellular serotonin abundance in layer 2/3 of the barrel cortex, which in turn strengthens the excitatory glutamatergic synaptic connections between the sensory input layer 4 and the cortical output layer 2/3, thus enhancing somatosensory output [11]. (C) Visual or whisker deprivation in mice results in reduced oxytocin secretion from the hypothalamus, which leads to reduced synaptic transmission to somatosensory or visual cortical output layer 2/3, reducing the output [13]. Rectangles represent sensory or sensory input neurons and ovals represent downstream output neurons.

Cross-modal compensation for sensory loss is an intriguing adaptive capacity, which has been studied so far in complex mammalian brains. Here we have shown that it also occurs in a considerably simpler animal, *C*. *elegans*, and is thus perhaps a fundamental feature of any nervous system. Our results suggest that decreased neuropeptide (including FLP-20) secretion from the TRNs in Mec mutants leads to an increase in the strength of inhibitory synaptic transmission between the AWC and AIY neurons in the olfactory circuit (Fig 8A), resulting in enhanced coupling between reversing frequency and food odor abundance, and in general, in increased olfactory acuity to AWC-sensed odors. Several lines of evidence support a causative link between these effects. First, loss of mechanosensation, and overall elimination of FLP-20 secretion cause a nonadditive increase in reversing rate upon removal from food (Fig 5A). Second, eliminating FLP-20 secretion exclusively from the TRNs, similarly, causes an increase in off-food reversing (Fig 5E). Third, such increased reversing can be counteracted by artificially and specifically attenuating AWC→AIY transmission (Fig 3D and 3E), demonstrating that changes in the strength of AWC→AIY transmission is sufficient to affect reversing rate. Moreover, reducing AWC→AIY transmission suppresses the increased reversal rates of *mec-4* and *flp-20* mutants (Fig 3F and Fig 6C). Fourth, increased reversing off-food is a key component of chemotaxis towards food, enabling reorientation in search for food when sensing a drop in food odor concentration [26–28]. Finally, AWC→AIY transmission, reversing rate, and chemotaxis are all modulated in a correlated fashion by FLP-20 signaling. Parsimony argues that these effects are likely to be linked, rather than separate actions of FLP-20 released, for example, independently from different neurons.

Our findings also hint at further possible components in this pathway. For example, additional TRN-secreted neuropeptides might be involved in signaling (e.g., Fig 5A); FLP-20 signaling itself might be amplified, for instance, by the ASE neurons (Fig 5H); and it is still not clear whether FLP-20 impact on AWC→AIY transmission is direct or indirect.

Recent studies have revealed a role for neuropeptide/hormonal signaling in cross-modal interactions in *C*. *elegans* during environmental stress [14,16] or developmental states of quiescence [58]. Our study indicates that in addition to conjoining simultaneous co-occurring inputs from different sensory modalities, neuropeptides can link between past mechanosensory experience and present chemosensory performance. Our findings also demonstrate that in addition to modulating sensory transduction at the sensory neuron level (e.g., tuning receptor strength), cross-modal neuropeptide signaling can also act at the circuit level, modifying the strength of synaptic transmission and downstream sensory processing. Furthermore, our study sheds light not only on the mechanisms of adaptation to sensory loss but also on normal sensory function, revealing an innate cross-network suppression mechanism. We speculate that cross-modal suppression by functional sensory neurons might serve to homeostatically limit to a manageable level the overall volume of sensory inputs that the nervous system receives, prioritizing diversity of sensory information over acuity of any one particular sensory modality. However, at the same time this mechanism is also flexible enough to enable the reweighting of sensory inputs in the event of sensory loss. Notably, the increased reversing rate in touch-deficient worms might have an additional advantage, as it effectively restricts their dispersal range, thus avoiding potential hazards that might otherwise be detected by a functional mechanosensory system.

In addition to enhanced chemosensation of AWC-sensed odors, cross-modal plasticity following mechanosensory loss appears to impact a range of behaviors, including reduced sensory responses to nose touch and to AWA-sensed attractants (Fig 1B and 1D). Whereas cross-modal enhanced sensory performance has obvious adaptive advantages, as it provides a form of compensation for unavailable sensory information, cross-modal reduced performance appears as maladaptive plasticity, whereby damage to one sensory modality propagates to diminish also other modalities. However, in some cases it might actually be beneficial. For example, worms withdrawing after being touched in the nose probably require body touch information to ensure that they don’t encounter another threat while reversing. When such information is permanently unavailable, it is perhaps more prudent to limit the response to nose touch as mechanosensory animals do (Fig 1B). Uncovering the intricate mechanisms underlying these additional forms of cross-modal plasticity and perhaps also their significance as an adaptive or maladaptive response to sensory deprivation is an appealing direction for future research.

In many ways the *C*. *elegans* cross-modal plasticity mechanism is analogous to cross-modal plasticity mechanisms underlying enhanced somatosensation in visually deprived rats (Fig 8B) [11,12] and reduced sensory processing in sensory-deprived neonatal mice (Fig 8C) [13], whereby the loss of one sensory modality leads to long-distance signaling (serotonin [11] or oxytocin [13]) affecting a second sensory modality through experience-dependent modification of key glutamatergic synapses in the sensory circuit (Fig 8). Specifically, our work reveals that the source for cross-modal signaling in touch-deprived *C*. *elegans* is the touch sensory neurons themselves (Fig 8A) rather than regions downstream of the sensory neurons in sensory signaling pathways (Fig 8B and 8C). Furthermore, it is reduced signaling (Fig 8A) rather than enhanced signaling (Fig 8B and 8C) that results in increased synaptic strength. Whereas the role of the sensory neurons themselves in this cross modal plasticity might be specific to the simple nervous system of *C*. *elegans*, the disinhibition by sensory deprivation, although not previously demonstrated, is likely to be conserved. Importantly, the various parallels that we have revealed between *C*. *elegans* and mice and rats support conservation of mechanisms identified in this study and the potential of using *C*. *elegans* for further research that will enhance our understanding of the molecular and cellular mechanisms governing cross-modal plasticity. Conversely, our findings generate specific predictions for mammalian systems. Namely, neuropeptides or other neuromodulators, originating from the sensory-deprived brain regions, might have a role in cross-modal plasticity both in normal and in sensory-deprived animals.

Optogenetics has been suggested as an approach for treatment of various brain diseases and malfunctions [59,60] such as epilepsy [61], stroke [62], and blindness [63]. We have shown that random optogenetic activation of the TRNs of Mec-deficient worms (Fig 8A, grey lightning bolt) could counteract the changes in locomotion resulting from the loss of mechanosensation. We have also demonstrated that locomotion can be restored by an alternative strategy, synaptic engineering. Inserting an electrical synapse between AWC and AIY that modifies AWC→AIY transmission (Fig 8A, grey dashed line), was sufficient to offset the enhanced reversing of Mec mutants. Thus, manipulating neuronal activity, by optogenetic stimulation, or artificially modifying synaptic transmission, by engineering new synapses into neural circuits, may potentially help recover deviations from normal circuit function.

## Materials and Methods

### *C*. *elegans* Strains

Strains were grown and maintained under standard conditions at 20°C on nematode growth medium (NGM) 2% agar plates seeded with *Escherichia coli* strain OP50. All experiments were conducted at 18°C–22°C. We found that higher temperatures considerably altered the results.

Wild-type worms were Bristol variety N2. The other strains used in this study are detailed in Table 1.

**Table 1:** List of strains used in the study.

Strain	Use	Figure
**TU253** *mec-4(u253)X*	Standard Mec mutant	1A–1G; 3A, 3B, 3F; 4B; 5A, 5E
**CB1339** *mec-4(e1339)X*	Mec allele	1E, 1F
**CB1515** *mec-10(e1515)X*	Mec mutant	1E, 1F
**BJH255** *mec-4(u253)X; ljIs111[Pmec-4*::*ChR2]*	TRN optogenetics	1H; 3C; 4C; 5F
**AQ2137** *ljIs109[Pnmr-1*::*YCD3*, *unc-119(+)]*	AVA/E imaging	2B
**AQ2207** *mec-4(u253)X; ljIs109[Pnmr-1*::*YCD3*, *unc-119(+)]*	AVA/E imaging	2B
**AQ2632** *ljEx351[Podr-1*::*YC3*.60*, *Pelt-2*::*mCherry]*	AWC imaging	2C; 6A
**MF290** *mec-4(u253)X*; *ljEx351[Podr-1*::*YC3*.60*, *Pelt-2*::*mCherry]*	AWC imaging	2C
**CX7569** *kyEx903[Podr-2b*::*Gcamp*, *Punc-122*::*GFP]*	AIB imaging	2D
**MF291** *mec-4(u253)X*; *kyEx903[Podr-2b*::*Gcamp*, *unc-122*::*GFP]*	AIB imaging	2D
**AQ2637** *ljEx354[Pttx-3(int2)*::*YC3*.60*]	AIY imaging	2E; 6B
**AQ2801** *mec-4(u253)X*; *ljEx354[Pttx-3(int2)*::*YC3*.60*]	AIY imaging	2E
**CX9193** *kyEx1844 [Pttx-3*::*glc-3cDNA*, *Pelt-2*::*gfp]*	AIY receptor overexpression	3A
**MF292** *mec-4(u253)X*;*kyEx1844 [Pttx-3*::*glc-3cDNA*, *Pelt-2*::*gfp]*	AIY receptor overexpression	3A
**RB594** *glc-3(ok321)V*	Receptor mutant	3B
**MF293** *mec-4(u253)X*; *glc-3(ok321)V*	Double mutant	3B
**BJH257** *mec-4(u253)X; glc-3(ok321)V; ljIs111[Pmec-4*::*ChR2]*	TRN optogenetics	3C
**AQ2614** *ljEx339[Podr-1*::Cx36*::*mCherry*, *Pttx-3(int2)*::Cx36*::*mCherry*, *Punc-122*::*mCherry]*	Synaptic engineering	3E
**BJH455** *mec-4(u253); ljEx339[Podr-1*::Cx36*::*mCherry*, *Pttx-3(int2)*::Cx36*::*mCherry*, *Punc-122*::*mCherry]*	Synaptic engineering	3F
**TU3568** *sid-1(pk3321)V; him-5(e1490)V; lin-15B(n744)X; uIs71[Pmec-18*::*sid-1*, *Pmyo-2*::*mCherry]*	TRN-specific RNAi	4A
**MF297** *egl-3(nr2090)*	Neuropeptide synthesis mutant	4B
**BJH259** *mec-4(u253)X; pekEx52[Pmec-4*::*EGL-21 sense*::*SL2*::*mCherry*, *Pmec-4*::*EGL-21 anti-sense*::*SL2*::*mCherry*, *Punc-122*::*mCherry]; ljIs111[Pmec-4*::*ChR2]*	TRN optogenetics	4C
**AX4239 ***dbEx708[Pmec-4*::*ins-1*::*mCherry]*	Coelomocyte assay	4D
**AX4240 ***mec-4*(*u253*)*X; dbEx708 [mec-4*::*ins-1*::*mCherry]*	Coelomocyte assay	4D
**PT505** *flp-20(pk1596)X*	FLP-20 mutant	5A, 5B; 6C, 6D
**MF294** *mec-4(u253)X; flp-20(pk1596)X*	Double mutant	5A
**RB2188** *flp-20(ok2964)X*	FLP-20 allele	5B, 5C, 5D
**BJH456** *flp-20(ok2964)X; ljEx806[Pmec-4*::*flp-20 cDNA*, *Punc-122*::*GFP]*	FLP-20 TRN rescue	5C, 5D
**BJH440** *pekSi28[cb-unc-119(+)*::*Pflp-20*::*FRT*::*FLP-20*::*SL2*::*mCherry*::*terminator*::*FRT*::*GFP]II*	FLP-20 TRN dysfunctional	5E
**BJH457** *pekSi28[cb-unc-119(+)*::*Pflp-20*::*FRT*::*FLP-20*::*SL2*::*mCherry*::*terminator*::*FRT*::*GFP]II; pekEx143[Pmec-4*::*FLP recombinase*, *Pelt-2*::*mCherry]*	FLP-20 TRN dysfunctional	5E
**MF298** *ynEx186[Pmec-7*::*flp-20(cDNA)*, *Pmyo-2*::*GFP]*	FLP-20 TRN overexpression	5F
**MF299** *mec-4(u253)X; ynEx186[Pmec-7*::*flp-20(cDNA)*, *Pmyo-2*::*GFP]*	FLP-20 TRN overexpression	5F
**BJH256** *mec-4(u253)X; flp-20(pk1596)X; ljIs111[Pmec-4*::*ChR2]*	TRN optogenetics	5G
*ynIs53[Pflp-20*::*GFP]*	FLP-20 expression	5H
*mec-4(u253)X; ynIs53 [Pflp-20*::*GFP]*	FLP-20 expression	5H
*pl5Ex5[Pmec-4*::*tetX*::*SL2*::*mCherry]; ynIs53 [Pflp-20*::*GFP]*	FLP-20 expression	5H
**MF300** *flp-20(pk1596)X*; *ljEx351[Podr-1*::*YC3*.60*, *Pelt-2*::*mCherry]*	AWC imaging	6A
**MF296** *flp-20(pk1596)X*; *ljEx354[Pttx-3(int2)*::*YC3*.60*]	AIY imaging	6B
**BJH458** *flp-20(pk1596)X; ljEx339[Podr-1*::Cx36*::*mCherry*, *Pttx-3(int2)*::Cx36*::*mCherry*, *Punc-122*::*mCherry]*	Synaptic engineering	6C

*Codon optimized

### Gentle Body Touch

A standard assay for gentle body touch was applied [43,44], whereby each worm was alternately touched five times anteriorly or posteriorly with an iris hair. For each worm, the number of anterior withdrawals was recorded.

### Chemotaxis

Chemotaxis assays were performed essentially as described [22]. However, we found that washing *mec-4(u253)* worms prior to the assay causes them to clump on the test plate. To overcome this, for Figs 1C and 6D, we manually transferred 20 worms to 6 cm unseeded nematode growth medium (NGM) test plates after releasing them from food on an empty plate. Prior to the assay, we placed 1 μL drop of 1% Bz diluted in ethanol on one side of the plate and a drop of ethanol alone on the opposite end. To each drop, 1 mM sodium azide was added for trapping the worms. This procedure was very noisy due to the relatively small number (*n* = 20) of worms in each plate. For low odor dilutions in Fig 1D, we did wash the worms and place them in a 9 cm test plate, but waited 15 min, the time it took *mec-4(u253)* to unclump, then applied the odor and the ethanol, waited another 5 min, and applied the sodium azide. In both cases chemotaxis was scored >2 h later by counting the number of paralyzed worms within the field of view of a stereomicroscope centered at the odor spot, *N(odor)*, and the number of paralyzed worms within the field of view centered at the control spot, *N(control)*, and calculating the chemotaxis index (CI) [22] equal to: [*N(odor)*-*N(control)*]/[*N(odor)*+*N(control)*].

### Reversing Rate

The reversing assay was performed as previously described [27]. A single worm was removed from food, allowed to crawl for a few seconds until no traces of food were visible in its track, and then transferred either to an empty 6 cm NGM plate (off-food assay) or to a 6 cm NGM plate seeded the day before with OP50 (on-food assay). After 1 min, reversing events consisting of at least one body bend were counted over a 3 min period.

### Slowing on Food

Speed off and on food was measured as in [31] by counting the number of body bends over a period of 20 s in the absence or presence of OP50 bacteria.

### Artificial Optogenetic TRN Stimulation

Worms were grown in the dark, and unless otherwise indicated, were fed OP50 bacteria mixed with ATR at 0.5 mM concentration. Just before blue light stimulation, 10 worms were transferred to a 3 cm plate whose lid was removed. For illumination, we used a Royal-Blue (447.5nm) LUXEON SR-03-R0500 Rebel LED assembly attached to a Carclo 27° Frosted 20 mm Circular Beam Optic (Part 10508; www.luxeonstar.com). The LED was controlled by an Arduino Uno R3 microcontroller (www.adafruit.com) using a Matlab (Mathworks) interface, which generated random blue light flashes for approximately 80 min each session. The interval between flashes was drawn from an exponential random distribution with a 10 s mean. The duration of each flash was drawn from a uniform distribution with a 3 s mean. Four to six worms were picked from the 3 cm plates either immediately (Fig 1H), after no flashing at all (Figs 3C, 4C and 5F), or 2 h after the end of the flashing session, and their reversing rate off food was measured. Data in Figs 4C and 5F are presented as the reversing rate after 2 h of TRN stimulation normalized by the average reversing rate without any stimulation for each experiment day.

### Calcium Imaging

Imaging was performed on a Zeiss Axioscope upright microscope equipped with a Hamamatsu ORCA-ER digital camera. Worms were inserted into a microfluidic PDMS chip [33], and the neuron of interest was observed through the chip's cover slip, using a 63X oil immersion objective. For imaging of spontaneous AVA/E activity, we used the “locomotion chip” [33], in which the worm is trapped, but is free to make forward and backward undulating movements similar to those observed during locomotion. Since the AVA and AVE neurons are very close and difficult to separate, we imaged them together as one unit. For imaging calcium responses to Bz removal in AWC, AIB, and AIY, we used the “olfactory chip” [33], which exposes the worm's nose to a constant flow of buffer (S-basal without cholesterol) or odor (unless otherwise mentioned, 1:10,000 Bz diluted in buffer). Imaging commenced after 5 min of odor exposure. 10 s after the beginning of each recording, the odor was replaced with buffer and the imaging continued for an additional 30 s. Movies were captured and analyzed using custom written Matlab (Mathworks) programs. A rectangular region of interest (ROI) was drawn surrounding the cell body (AVA/E, AWC, and AIB) or the neurite (AIY) [28], and for every frame the ROI was automatically shifted according to the new position of the center of mass (in the case of the cell body) or point of maximum intensity (AIY neural process). The fluorescence intensity, F, was computed as the difference between the sum of pixel intensities and the faintest 10% pixels (background) within the ROI. For ratiometric imaging (all neurons except for AIB), only ROI_YFP_ was tracked, whereas ROI_CFP_ remained at a fixed offset from ROI_YFP_. The ratio, R, between F_YFP_ and F_CFP_ was then computed after correcting for bleed through. No correction for bleaching was necessary. Spontaneous calcium transients in AVA/E were detected automatically. All transients in a recording were aligned and averaged, and then all averaged traces were averaged between worms to obtain an overall mean trace. The traces in the olfactory imaging experiments depicting ΔF (AIB) or ΔR (AWC and AIY), were computed as (F − F_0_) / F_0_ * 100, whereby F_0_ equals the average F within the first 3 s of recording. For statistical quantification, ΔF (and similarly ΔR) was computed as (F_1_ − F_0_) / F_0_ * 100, whereby F_0_ is the average F over T_0_, the 10 s preceding odor switching, and F_1_ is the average F over T_1_, the 10 sec following odor switching (see Fig 2D). For AIB, (F_2_ − F_0_) / F_0_ * 100 was computed as well, F_2_ being the average F over T_2_, the last 10 s of the recording. All imaging strains showed normal reversing behavior.

### Synaptic Engineering of AWC-AIY Electrical Synapse

We used a strain previously described, AQ2614 [42], containing an electrical synapse inserted between AWC and AIY. Briefly, the cDNA sequence of *Mus musculus* gap junction protein, delta 2 (Gjd2), was codon optimized to produce a synthetic Cx36 gene (GeneArt). We fused to Cx36 an upstream promoter (either P*odr-1* for AWC [28] or Pttx-3(int2), the second intron of the *ttx-3* gene, for AIY [64]) and a downstream gene encoding mCherry, and coinjected both plasmids to generate a transgenic worm carrying an extrachromosomal array.

### TRN-Specific RNAi Knockdown

TRN-specific RNA interference (RNAi) by feeding [43] was performed as follows. Worms from the TU3568 strain, *sid-1(pk3321)V; him-5(e1490)V; lin-15B(n744)X; uIs71[Pmec-18*::*sid-1*, *Pmyo-2*::*mCherry]*, were fed with bacteria either carrying an empty vector (control) or producing double-stranded RNA against *mec-4* (Source BioScienceRNAi library, ID number X-7D15; primer pairs: forward, TACCTGCAACGGAAAGATCC and reverse, ATACAACGGAAAGACGCCAC) or *egl-3* (Source BioScienceRNAi library, ID number V-7G01; primer pairs: forward, CATGAATGCATTACTCACTTGGA and reverse, CATATCTACTCTGCTTCATGGGG) [65]. To prepare the bacteria, a single colony of each bacterial strain was shaken overnight at 25°C in LB medium containing 50 μg/ml ampicillin and 12.5 μg/ml tetracyclin. Then 25 ml of LB medium containing 50 μg/ml ampicillin were inoculated with 250 μl culture and shaken for 4–5 h at 37°C until a 595 nm absorbance of at least 0.8 was reached. Next, an additional 25 ml of LB medium containing 50 μg/ml ampicillin was added to the culture together with 1M IPTG. Culture was shaken at 37°C for an additional 4 h and then spun down. The precipitate was resuspended in 2 ml M9 buffer containing 10 μl 1M IPTG and distributed onto NGM plates supplemented with 25 μg/ml carbenicillin.

TRN-specific RNA interference (RNAi) by transgenic transformation [45] was performed by coinjecting plasmids containing 738 base pair sense and antisense fragments from the coding region of the *egl-21* gene fused to the mec-4 promoter into *mec-4(u253)* worms.

### Neuropeptide Secretion

To gauge overall neuropeptide secretion, we imaged the coelomocytes of wild type and *mec-4(u253)* mutants specifically expressing in their TRNs (using a *mec-4* promoter) INS-1 fused to mCherry. Transgenic animals were picked at the L4 stage onto fresh plates and were grown for an additional 12–16 h before being treated with 0.01 M of the anesthetic sodium azide (NaN3) and then imaged. We examined only animals oriented laterally on their right side and accordingly imaged ccDR, ccAR, and ccPR coelomocytes. Fluorescence intensity was determined after background subtraction using Metamorph software. As the difference between wild type and *mec-4* coelomocyte fluorescence intensity was similar in anterior and posterior coelomocytes (S1B Fig), we pooled them for analysis.

To monitor FLP-20 transcription, we recorded fluorescence intensity in ALM, PVC, and ASE neurons of a Pflp-20::GFP integrated strain, similarly to the coelomocyte fluorescence measurement described above. The Pflp-20::GFP-integrated strain carrying the extrachromosomal array *pl5Ex5[Pmec-4*::*tetX*::*SL2*::*mCherry]* was imaged with both GFP and RFP channels. We analysed only animals expressing RFP in both ALM and PLM. We controlled *mec-4* promoter transcriptional activity by measuring the fluorescence intensity of Pmec-4 driven YC2.12 in wild type and *mec-4(u253)* mutants treated with 0.01 M sodium azide. We found no significant difference in YFP expression between them (S1A Fig).

### TRN-Specific Dysfunctional FLP-20

We made a Gateway pENTR221 vector, BJP-I135, including FRT-flanked FLP-20 genomic DNA fused to SL2, mCherry, and a terminator, let-858 3’ UTR, by modifying the plasmid pWD178 [66], a gift from the Jorgensen lab. We used Gateway to recombine Pflp-20 upstream and GFP downstream of the construct into a MosSCI destination vector pCFJ150 [52], targeting a ttTi5605 Mos1 insertion on chromosome II. We then generated a single copy insertion of this cassette and crossed it into *flp-20(pk1596)* to obtain the rescue strain BJH440. We injected into BJH440 Pmec-4::FLP recombinase and obtained BJH457. In this strain, FLP recombinase excises the FLP-20::SL2::mCherry sequence specifically in the TRNs. As a result, all neurons expressing FLP-20 have a functional copy of the gene, except for the TRNs.

### In Vitro Receptor-Ligand Binding Assay

Chinese hamster ovary (CHO) cells K1 stably overexpressing the mitochondrial targeted apo-aequorin (mtAEQ) and the human Gα16 subunit were cultured and transfected with pcDNA3.1D::receptor-cDNA as described fully in [57]. Cells for negative control experiments were transfected with an empty pcDNA3.1D vector. The three FLP-20 peptides, (AMMRFamide, AVFRMamide and SVFRLamide) were synthesised by Cambridge Research Biochemicals, based on in silico predictions. All peptides were initially tested at a concentration of 10–5 M. In addition, BSA medium containing no peptides was used as a negative control, and ATP, which activates an endogenous CHO receptor, was used as a positive control. BSA and ATP responses were averaged for all transfected constructs. Calcium responses were monitored as previously described [57] for 30 s on a Mithras LB 940 luminometer (Berthold Technologies).

The numerical data used in all figures are included in S1 Data.

## Supporting Information

S1 Data Raw numerical data for figure panels. 1A, 1B, 1C, 1D, 1E, 1F, 1G, 1H, 2B, 2C, 2D, 2E, 3A, 3B, 3C, 3E, 3F, 4A, 4B, 4C, 4D, 5A, 5B, 5C, 5D, 5E, 5F, 5G, 5H, 6A, 6B, 6C, 6D, 7, S1A Fig, S1B Fig.
(XLSX)

S1 Fig Touch receptor neurons and coelomocyte fluorescence. (A) YFP fluorescence in touch receptor neuron ALM of wild type (strain **AQ906** *bzis17[Pmec-4*::*yc2*.*12*, *lin-15(+)]*) and *mec-4* mutants (strain **AQ908** *mec-4(u253); bzis17[Pmec-4*::*yc2*.*12*, *lin-15(+)]*). (B) Anterior and posterior coelomocyte fluorescence in wild type (strain **AX4239** *dbEx708[Pmec-4*::*ins-1*::*mCherry]*) and *mec-4* mutants (strain **AX4240** *mec-4*(*u253*)*X; dbEx708 [mec-4*::*ins-1*::*mCherry]*).
(TIF)

## Abbreviations

AMPA: α-amino-3-hydroxy-5-methyl-4-isoxazolepropionic acid
ATR: all-trans retinal
BSA: bovine serum albumin
Bz: benzaldehyde
CHO: Chinese hamster ovary
ChR2: Channelrhodopsin2
DA: diacetyl
IAA: isoamyl alcohol
INS-1: insulin-like peptide transgene
Mec: mechanosensory
MosSCI: Mos1 single-copy insertion
Py: pyrazine
SEM: standard error of the mean
TRN: touch receptor neuron
