# Supplementary figures and images for: Neuropeptide-Driven Cross-Modal Plasticity following Sensory Loss in Caenorhabditis elegans
Source: PLoS Biol. 2016 Jan 8;14(1):e1002348. doi: 10.1371/journal.pbio.1002348 (PMC4712962; doi:10.1371/journal.pbio.1002348)

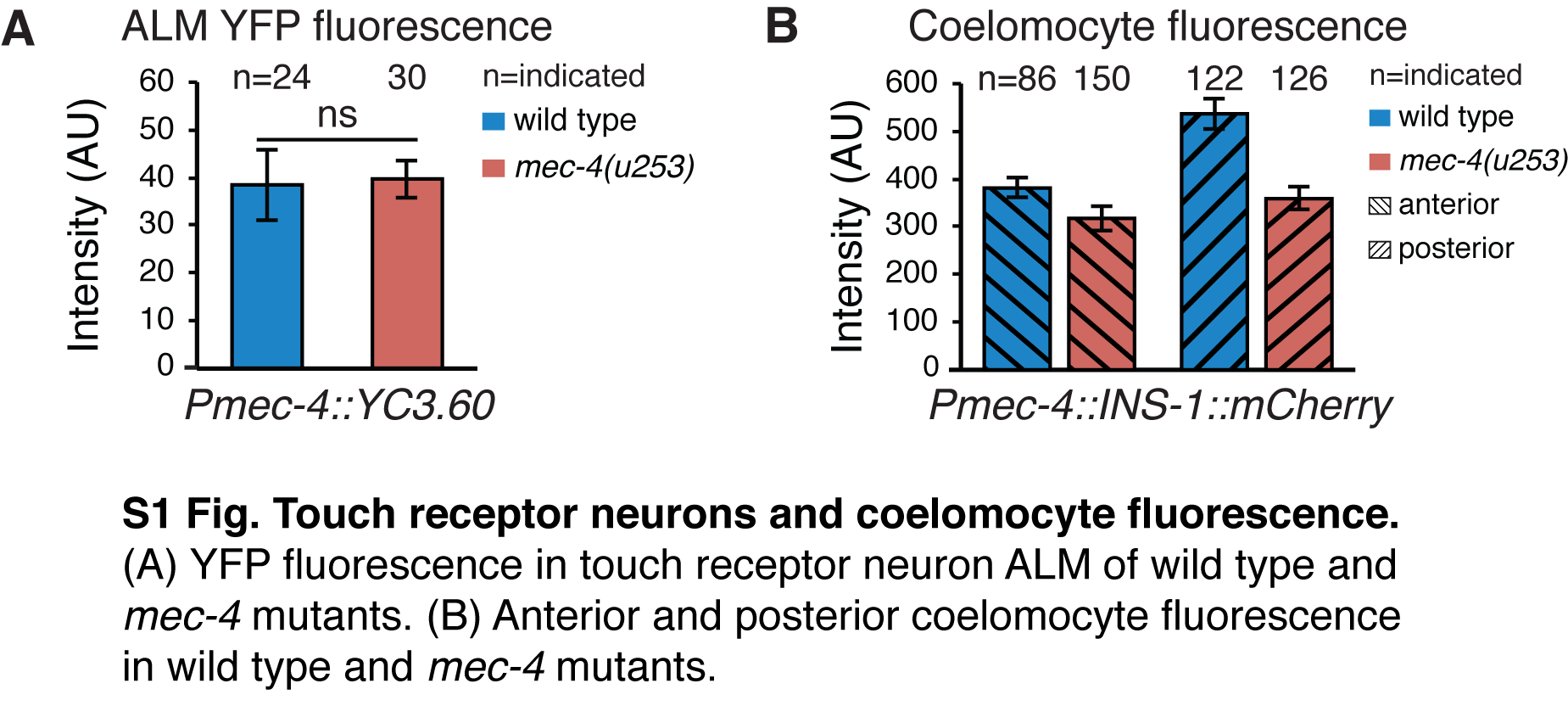

Supplement: S1 Fig — (A) YFP fluorescence in touch receptor neuron ALM of wild type (strain AQ906 bzis17[Pmec-4::yc2.12, lin-15(+)]) and mec-4 mutants (strain AQ908 mec-4(u253); bzis17[Pmec-4::yc2.12, lin-15(+)]). (B) Anterior and posterior coelomocyte fluorescence in wild type (strain AX4239 dbEx708[Pmec-4::ins-1::mCherry]) and mec-4 mutants (strain AX4240 mec-4(u253)X; dbEx708 [mec-4::ins-1::mCherry]). (TIF) [file pbio.1002348.s002.tif]
